# Challenges in eating disorder diagnosis and management among family physicians and trainees: a qualitative study

**DOI:** 10.1186/s40337-022-00570-5

**Published:** 2022-03-31

**Authors:** Angel Tse, Sabatinie Xavier, Karen Trollope-Kumar, Gina Agarwal, Cynthia Lokker

**Affiliations:** 1 Department of Family Medicine, University of British Columbia, Vancouver, BC Canada; 2 McMaster University, Hamilton, ON Canada; 3 Department of Family Medicine, McMaster University, Hamilton, ON Canada; 4 Body Brave, 1047 Main Street East, Hamilton, ON Canada; 5 Health Information Research Unit, Department of Health Research Methods, Evidence and Impact, McMaster University, CRL 137, 1280 Main St W, Hamilton, ON L8S 4K1 Canada

**Keywords:** Feeding and eating disorders, Internship and residency, Medical education, Primary care, Family medicine, Occupational stress, Moral distress, Physician burnout

## Abstract

**Background:**

Family physicians are one of the first points of contact for individuals with eating disorders (EDs) seeking care and treatment, but training in this area is suboptimal and insufficient. Specialized ED treatment programs often have long wait lists, and family physicians are responsible for patients care in the interim. The aim of this study was to identify the learning needs and challenges faced by Canadian family physicians and trainees when caring for patients with EDs.

**Methods:**

We recruited six family medicine residents and five family physicians practicing in an academic unit in the Department of Family Medicine of a medical school in urban southwestern Ontario, Canada. We used purposive sampling, focusing on residents and faculty physicians from the department and conducted one focus group for the residents and another for the faculty physicians, exploring their clinical knowledge and challenges when managing ED patients. The focus groups were audio-recorded and transcribed verbatim prior to thematic coding.

**Results:**

Physicians and residents faced challenges in discussing, screening, and managing patients with EDs. Three themes that emerged from the qualitative data highlighted training needs related to: (a) improving communication skills when treating a patient with an ED, (b) more effective screening and diagnosis in primary care practice, and (c) optimizing management strategies for patients with an ED, especially patients who are waiting for more intensive treatment. A fourth theme that emerged was the distress experienced by family physicians as they try best to manage and access care for their patients with EDs.

**Conclusion:**

Addressing the learning needs identified in this study through continuing education offerings could aid family physicians in confidently providing effective, evidence-based care to patients with EDs. Improvement in training and education could also alleviate some of the distress faced by family physicians in managing patients with EDs. Ultimately, system changes to allow more efficient and appropriate levels of care for patients with EDs, removing the burden from family medicine, are critical as EDs are on the rise.

**Plain English summary:**

A person with an eating disorder will normally seek care from their family physician first. These conditions can dramatically reduce the quality of a person’s life and health. Family physicians therefore need to know how best to help these patients or refer them to a more intensive level of care, which often has long wait lists. We asked a group of family physicians and a group of family medicine trainees about their experiences with patients with eating disorders and about the information they wished they had to help these patients. The results show that they need more information on how to talk to a patient about eating disorders without judgement, how to diagnose a patient with an eating disorder, and then what treatment and management is needed while they wait for more intensive treatment for sicker patients. The physicians and trainees both talked about the stress and worry that they faced when treating patients with eating disorders. Besides their lack of training about these conditions, family physicians also described difficulties when trying to access timely specialized services for their patients. Physicians can experience moral distress when they know that their patients need higher level care, but there are systemic barriers to specialized programs that block their patients from getting the care they need when they need it.

**Supplementary Information:**

The online version contains supplementary material available at 10.1186/s40337-022-00570-5.

## Background

In Canada, an estimated 2.9 million people meet the diagnostic criteria for an eating disorder (ED) [1]. EDs are serious psychiatric conditions characterized by significant disturbances in behaviours and attitudes that surround eating, body weight, and body shape [2]. Among mental illnesses, EDs have a high mortality rate, second only to the opioid crisis [3].

Many individuals struggling with an ED will first consult their family physicians who will frequently be responsible for their ongoing care due to the shortage of publicly funded ED clinics and treatment centers for adult or pediatric patients [4–7]. The role of the family physician ranges from prevention, detection of risk factors, diagnosis, determining the need for more advanced treatment (e.g., specialist referrals or hospitalization), and managing medical complications [8]. Knowledge gaps about diagnosis and treatment of EDs have been identified for a range of healthcare providers [9] and pose a significant problem since early intervention maximizes the chances of recovery [10].

Training for professionals involved in the care and treatment of EDs is suboptimal [7, 11–13], which can result in patients with EDs not receiving appropriate evidence-based psychosocial interventions [10]. In the USA, 80% of surveyed residency programs did not offer ED rotations [14]. Canadian medical students receive only 3–5 h of education about EDs in their undergraduate years, and many do not treat patients with EDs in a clinical placement. A survey of family physicians and psychologists in Ontario identified need for increased training and support regarding screening, multi-informant assessment methods, evidence-based interventions [11, 15] and parental involvement with pediatric and adolescent patients [15].

## Methods

The objective of this study was to identify the learning needs and challenges faced by family physicians and trainees when caring for their patients with EDs.

### Study design

We used a qualitative approach including two focus groups to allow for an in-depth understanding of the perceptions and challenges regarding the ongoing assessment, treatment, and care of patients with EDs by family physicians and trainees. Qualitative approaches enable the collection of rich information where discussions can emerge and progress naturally.

After receiving ethics approval from the Hamilton Integrated Research Ethics Board (HiREB-7329), we recruited a purposive sample of family medicine residents and practicing family physicians who are faculty members in the Department of Family Medicine at McMaster University. All were eligible for inclusion regardless of age, sex, gender, or ethnicity. We recruited through the McMaster Department of Family Medicine faculty and residents list.

Two in-person focus groups, with family physicians and trainees respectively, were held, each 90 min in duration, and followed semi-structured questions about training experience and identification of relevant topics [16]. We identified relevant topics to discuss based on the services that family physicians most often provide to ED patients, such as diagnosis, symptom management, referrals, educational information, and counseling [12]. We asked participants to introduce themselves and share clinical experiences that highlighted their knowledge needs. The sessions followed the semi-structured guide with additional probing and follow up questions to get further details. Participants completed an informed consent form and demographic questionnaire before participating in the study and received an honorarium in appreciation for their time.

We collected data between October 2019 and February 2020. The guide containing questions about family physician training experience is provided in Additional file 1: Appendix. The focus groups were facilitated by KTK, a family medicine physician for 35 years who has extensive experience in qualitative research and is the director of clinical services for Body Brave, a non-profit community organization that provides services for people with EDs. Study team members observed and took notes (CL, SX, AT). Focus groups and individual interviews were audio recorded and transcribed verbatim with identifying information removed.

We used thematic analysis based on Braun and Clarke’s six phases of analysis to analyze the data and group related codes into semantic themes [17, 18]. Themes were identified based on the explicit meanings of the data provided by family physicians and trainees [17]. The first phase involved reading through the transcribed data and notes taken during the interviews and focus groups; two research assistants (SX, AT) reviewed the data for accuracy. In phase two, the research assistants developed initial data-driven codes based on the transcripts and notes taken during the focus groups and interviews [17]. The two research assistants (an undergraduate thesis student and a medical student, both with informal training in qualitative data analysis) independently coded the focus group transcripts to increase credibility while identifying findings relevant to our research question. We discussed the duplicate coding to determine alignment, disagreements, and overlap in the independent codes. In phrase three, we searched for general themes in the coded data [17]; we developed the coding structure for the data and coded the transcripts independently using NVivo. In phase four, we reviewed the themes for coherence in patterns [17]. In phase five, we grouped smaller themes and labeled them based on broader, overarching themes. New codes that emerged from each transcript were identified and discussed within the research team to ensure agreement. For additional external validation, we discussed the process of analysis and major themes with a qualitative researcher not involved in the analysis process (GA). This increased the rigor of our analysis. In phase six, we chose vivid examples of the themes by presenting direct quotes from the participants in our report [17].

## Results

Six residents in a 2-year family medicine residency program and five practicing academic family physicians, with a range of time in residency and in practice, participated in two separate focus groups (Table 1). From the transcripts, we identified that insufficient education and training on EDs leads to family physicians/residents facing challenges in three major areas: communication, screening and diagnosis, and management of patients with EDs. A fourth theme, woven through all the transcripts, was the stress experienced by family physicians faced with managing patients with serious EDs when access to specialized treatment was lacking. The themes identified in the resident focus group were the same as the physicians.

**Table 1:** Characteristics of study participants

Type	Gender		Residency		Independent Practice		
	Male	Female	Year 1	Year 2	< 10 years	10–20 years	> 20 years
Residents	1	5	2	4	n/a	n/a	n/a
Family physicians	2	3	n/a	n/a	2	2	1

### Communication

Participants emphasized the need for communication strategies to help them appropriately engage patients when they suspect an ED. Patients may not be forthcoming about their illness due to stigma and/or societal idealization of thinness, strict dieting, and intense exercise routines. One physician shared an anecdote about a patient who took almost 20 years to reveal that she had an ED. Another physician followed up with this reflection:

I think that there is a fair degree of, uh, stigma involved with eating disorders... Most of the time people, uh, rather keep it quiet and suffer the consequences of the eating disorder…

Physicians also admitted hesitation in approaching the subject of EDs with their patients, and they shared instances of recognizing in hindsight how they allowed stereotypical portrayals of EDs to hinder their objective assessment of a patient. This consequently led to overlooking or downplaying questionable patient diets and bodily perceptions.

One was a case that made me look at my own bias because I had a patient who came in and told me that she was vomiting and binging and purging, but her weight was fine...it took me a while to really believe that she had an eating disorder.

Even with the awareness that EDs, like many mental health conditions, are an inherently sensitive topic, physicians expressed uncertainty on how to appropriately engage their patients. This uncertainty hinders their ability to provide high quality care as they are not comfortable discussing ED experiences. Participants thus emphasized a need for communication strategies specific to EDs.

I worry about bringing something up and then labelling somebody and then them not wanting to talk about it. So, I think maybe subconsciously I am avoiding it at times, which is terrible - right? Because we want to pick it up- so, that's what I struggle with.

### Screening and diagnosis

Participants expressed difficulty in screening patients and knowing what screening criteria they should be aware of.

I feel like I may be missing people, especially adolescents. So, thinking of different ways of screening my patients or if I'm having suspicions, knowing different strategies that I could use would be very helpful.

Several participants mentioned how difficult it can be to identify disordered eating patterns in patients who do not show the stereotypical signs and symptoms of an ED.

I think a lot of us are taught anorexia as not eating... and then bulimia and that's pretty much it. But there is a spectrum to the condition that I think we should be a little bit more aware of.

Participants shared instances of observing suspicious signs and symptoms but due to uncertainty, missed the opportunity to screen for an ED.

When people come to see me with a mental health issue, if it [ED] is not something on the horizon, it might be missed. What are the distinct clues to look for during an interview?

Family medicine residents also explored the specific case of considering EDs in patients seeking or recovering from bariatric surgery as one example of a red flag that is not often considered. Invasive weight-loss surgeries implement a physical change in the body to restrict food intake. However, if it is not maintained by changes in eating behaviour, patients suffer from physical repercussions such as the gastric pouch re-expanding. If a patient is suffering from an underlying ED that is not recognized, their clinical course following bariatric surgery can be complicated, particularly since many bariatric clinics do not do long-term follow-up. A resident completing a general surgery rotation noted:

I did work on bariatric services and we had a lot of patients who...regained all the weight because [their ED] wasn't addressed.

### Management and referrals

Participants discussed uncertainty on how to appropriately manage and monitor patients. This would include how frequently to conduct tests, what should be covered during physical check-ups, and how to monitor their patients’ emotional or mental well-being. Participants also expressed the need for more evidence-based approaches in managing patients, particularly when there are co-morbidities such as anxiety, depression, or diabetes. They wanted to learn about the safety of medications in ED treatment. They consistently brought up the need for guidance on how to provide emotional and psychological support, particularly when patients are on waitlists for specialized programs.

I think skills to do some counselling too...you know, I tried to use my support framework within those [ED] conditions but I don't think that's enough. I think we need to have specific skills for patients with eating disorders that I can use when I see them today.

Participants struggled with assessing their patient's safety when engaging in elective activities. They identified a need to learn which activities are permissible and which activities should be avoided at certain stages of their patient’s ED. Notably, participation in athletic activities, which is identified as a trigger for EDs, was a major area of doubt.

I think about whether they should still be allowed to do their activities, but I don't know when exactly I should be saying, “you should stop physical activity”, and when it's okay to say to start again.

After identifying patients with an ED and providing care, family physicians described challenges in referring them to specialized care due to a shortage in services, as well as financial and geographical barriers. Consequently, family physicians become responsible for complex ED cases out of their scope of practice.

We've been in a case recently with a patient in my practice where they were too sick for outpatient, they weren't sick enough for inpatient......those patients fall through the cracks- who sees them, who takes care of them? It's us, but we haven't had the training to be able to do it.

One participant reflected on the uncertainty they faced when navigating their role in providing ED care:

What is your role as the family doctor? You are identifying it; you are doing the physical part and the electrolyte part. But then there's also the psychotherapy part and that is the huge part of the treatment. That's probably the most challenging part and I think I feel unequipped.

### Physician distress

The participants in our study experienced significant stress in caring for their patients with EDs. Because of their lack of training, they described diminished self-confidence in diagnosis and management of patients with these conditions. Also, they expressed struggles with trying to obtain specialized treatment for their patients with EDs since wait lists for hospital-based services in the region are long, leaving them alone to care for their patients. One resident explained how frightening it is to see a person with an ED appear on the schedule:

I really panicked when I see them coming up on my schedule because I was like, I don't know how to approach this problem.

The faculty physicians were equally distressed about their lack of confidence in managing patients with EDs:

I think for me one of the most stressful things is when there's someone I'm actually worried about. I mean they're- they're stable in the sense that they don't qualify for an immediate emergency admission, um, but they're sick and you're worried and the outpatient clinic say they're too sick for us… it's that scary in-between time when people are on waitlists for beds, um, on waitlists to get into programs and they're sick.

Another faculty physician described having to manage a very sick patient without support:

I just feel kind of helpless… physician education and training for us in primary care will be super helpful to know what to do, rather than just cruise control with the monitoring, making sure that they're not imminently, you know, in that moment at significant risk.

## Discussion

### Learning needs and challenges

This Canadian study highlighted that family physicians and trainees face challenges with communication, screening and diagnosis, and management of EDs which aligns with a systematic review from 2017 that reported a lack of knowledge of diagnosis and management of EDs across a range of healthcare providers [9]. These needs stem from insufficient education and training during medical school and residency [12, 13, 16] and the need for accessible supports and additional education has been highlighted before [4, 7, 13, 19–21]. Participants discussed relying on external resources such as conferences, online searches, or help from allied health professionals when managing patients with an ED.

Recent research indicates that the prevalence of EDs is much higher than previous estimates, and these conditions are consistently underdiagnosed [1]. EDs can present with a broad range of symptoms in which diagnostic categories can be blurred, yet the formal education of our participants included only classical presentations of EDs which fails to prepare clinicians for identifying atypical or borderline signs and symptoms. Another effect of insufficient training and education was highlighted by participants who noted that their patients felt a lack of confidence in family physicians to recognize their ED and provide adequate care. In our study, a key challenge identified by family physicians was a need for comprehensive communication strategies. Due to the sensitive nature of mental illnesses, trust within a physician–patient relationship is paramount [22]. Participants want guidance on how to make patients feel comfortable and safe in their care. Establishing a supportive relationship with patients can reduce tendencies to hide or falsely report disordered eating habits; good doctor-patient relationships enable patients to share pertinent information for accurate diagnoses, follow advice, adhere to the prescribed treatment, and lead to better patient outcomes [23].

This echoes a study focusing on binge eating disorder which discerned a need for education on effective patient-physician dialogue that emphasized disorder specific diagnostic criteria and assessment of emotional relationship to body weight, food, and binge eating disorder [24]. Training physicians on appropriate versus inappropriate comments regarding diet and weight may reduce the occurrence of physicians portraying personal biases, overlooking nuanced ED presentations, and/or reluctance to discuss EDs [9, 24]. Physician understanding of stigma around EDs is necessary considering the weight biases that exist in healthcare and the negative effect of stigma on psychological triggers and eating pathology in patients [25]. For instance, physicians have observed colleagues having negative attitudes and making negative comments about obese patients; this strong weight bias increases the likelihood of attributing obesity to behavioural causes (e.g., lack of willpower) and frustration toward patients, which may affect treatment decisions [25]. The review by Seah et al. [9] indicated that many healthcare professionals view patients with EDs negatively. Supplementary training for family physicians should include methods to recognize societal biases and address personal perceptions concerning diet and weight.

As a family physician is the first point of contact for a patient with a medical condition, screening for EDs is crucial to early intervention and better treatment outcomes [26] yet is seldom done [27]. Due to limited time during appointments, participants said that EDs are often not considered, and they rarely perform screening. This finding is consistent with a survey that showed only 25% of family physicians routinely screen for EDs [11]. The need for more training on red flags, signs, and symptoms to prompt screening was highlighted by our participants. Similarly, the majority of surveyed frontline medical providers reported that they were unsure of how to screen for or treat EDs; they supported universal screening for EDs while citing the need for increased training to overcome ineffective screening and fear of incompetence [7].

Our participants repeatedly expressed their uncertainty about treating patients with EDs. They were not sure how to monitor patients’ physical status and how frequently to schedule follow-up appointments. They expressed difficulty in counselling on patient safety, particularly when to stop or resume physical activity, and uncertainty on managing patients with comorbidities. Furthermore, most treatments for EDs involve nutritional rehabilitation, psychiatric medications, and psychotherapy which our participants felt is beyond the scope of their practice [28]. Ultimately, they identified a need for evidence-based treatment protocols appropriate to a family practice setting, and these should incorporate interdisciplinary programming [12].

### Physician distress

Besides the stress associated with their gaps in knowledge, family physicians in our study also identified challenges in accessing specialized ED care for their patients. Programs for specialized ED treatment in Canada are limited in number, located in large urban centers. and have long waiting lists. Since the COVID-19 pandemic began, the reported prevalence of EDs has risen dramatically [29]. At some centers, staff from ED treatment programs were deployed to other areas of the hospital to address COVID-19-related needs. Waiting lists for treatment programs have lengthened from months to years, in many cases. Some specialized treatment programs for ED are covered by government health insurance; however, wait lists for these services are extremely long. Other hospital-based programs are partially subsidized by the government, but patients still face steep costs. Private treatment services are unaffordable for many Canadians.

Family physicians and trainees in our study expressed feelings of helplessness when responsible for patients who were unable to receive specialized care in a timely manner. These findings suggest that our participants could be experiencing significant moral distress as they face seemingly intractable systemic barriers to care. Moral distress has been described as the inability of a moral agent to act according to their core values and perceived obligations due to internal and external constraints [30]. Moral distress is increasingly recognized as a major factor in physician burnout [31], and caring for patients with EDs is also a factor affecting physician burnout [32].

Although the focus groups were conducted just prior to the onset of the COVID-19 pandemic, the stressors identified by our respondents have undoubtedly been exacerbated by the rise in ED prevalence and the lengthening of waiting lists for specialized treatment. Physician burnout has also reached unprecedented levels. A recent survey by the Ontario Medical Association revealed that 72% of doctors reported some symptoms of burnout, while 35% said that they were experiencing persistent or severe symptoms of burnout [33]. Addressing the issue of physician burnout is complex and multifaceted. In the specific case of family physicians treating EDs, training and support should include burnout management [32] but, in the bigger picture, systemic changes that include significantly more resources being allocated to ED treatment are needed.

In Canada, treatment for patients with EDs is still primarily delivered through hospital-based programs. Evidence supports a stepped care approach as an appropriate [34] and cost-effective way to treat people with EDs [35, 36]. Such stepped-care strategies for ED treatment offer great promise, ensuring that patients receive the right level of care at the right time. In Australia, community-based low intensity treatment programs are being rapidly rolled out across the country, providing accessible care for people with mild to moderate EDs [35]. Integrating such programs of care could reduce the distress faced by Canadian family physicians who are unable to obtain appropriate care for their patients with EDs in a timely manner while ensuring that specialized hospital-based treatment programs be reserved for patients with the most severe EDs.

### Limitations

The relatively small sample size and purposive sample of physicians could have led to a biased representation. As this study only includes family physicians in Ontario, the generalizability of the findings to other jurisdictions or specialties might be limited. Our purposive sample was drawn from residents and faculty members in an academic family medicine teaching unit, which could affect generalizability. Furthermore, pressures of social conformity and intellectualized responses in the focus groups should be considered.

The focus groups were facilitated by the lead investigator who has a background in family medicine, qualitative research, and experience with caring for patients with EDs. Being the cofounder of a not-for-profit that provides community-based care for people with EDs, and having engaged in medical education, she has a deep understanding of the issues facing educators, learners, and practicing clinicians. To increase reflexivity, the focus group questions were designed using peer-reviewed research and with the broader research team; the data analysis was completed independently, with themes presented and discussed with the broader research team. We acknowledge that our own subjectivity may have impacted the question topics, generation of codes, and some interpretation of data. Our aim was to thematically analyze the data and not to reach saturation. Further focus groups, across other settings, would improve generalizability and potential for saturation in themes.

## Conclusion

This study emphasized the need for improved training and support about effective communication strategies, screening and diagnosis, and management strategies that include specific details on what and how often to monitor patients with EDs for Canadian family physicians. With additional training, family physicians would feel more confident in recognizing and treating people with EDs in their practice. The shortage of specialized ED care particularly causes distress as family physicians care for patients quite ill with EDs. Better training and better access to appropriate treatment programs could improve patient outcomes for the millions of Canadians living with an ED and support the physicians who care for them.

## Supplementary Information

**Additional file 1.** Semi-structured question guide used during the focus groups.

## Data Availability

The datasets generated and/or analysed during the current study are not publicly available due the small sample size but may be available from the corresponding author on reasonable request.

## Abbreviation

ED: Eating disorder
