# Supplementary material for: Challenges in eating disorder diagnosis and management among family physicians and trainees: a qualitative study
Source: J Eat Disord. 2022 Mar 31;10:45. doi: 10.1186/s40337-022-00570-5 (PMC8968091; doi:10.1186/s40337-022-00570-5)
Supplement: Supplementary file 1 — Additional file 1. Semi-structured question guide used during the focus groups. [file 40337_2022_570_MOESM1_ESM.docx]

# Appendix

# Learning needs of family physicians and trainees on eating disorders

### Focus group topic guide

We’ll begin the session with introductions and rationale for the study. To get a better sense of participant’s experience, they will be asked to introduce themselves, tell us why they are interested in EDs, and how long they have been training or practicing. Participants will compete a survey of demographic data (years of training/practice, gender, age group (by decades).

Training experience

What sort of training have you already received regarding eating disorders? (This can include undergraduate or post-graduate training, short courses, professional development courses, etc) (We will have everyone answer this question)

Are you aware of any training that is available?

Training topics

Which topics would be helpful in an ED training course for GPs? Why?

Which topics would not be helpful or relevant in an ED training course for GPs? Why not?

Here’s a list of potential topics that are discussed in the literature. Are there any topics haven’t been discussed that you think should be included in an ED training course for GPs?

- - Screening and early detection
  - Prevention
  - Presenting complaints
  - Evidence-based treatments
  - Outpatient services
  - Resources for patients and caregivers
  - ED in pediatric and adolescent patients
  - Family involvement in assessment
  - Family involvement in treatment

Can you share a clinical experience that highlighted your knowledge needs?

Preferred course format/time/delivery

How might you envision this content being taught or presented?

If this was taught online, what topics might be suitable and how would you envision these being taught in an online format?

What is your preferred format for online learning? (prompts if needed: videos, case presentations, self-assessments, etc)

Are there elements to online learning that are not helpful or preferred? Do you have any suggestions for overcoming these?

Are case discussions helpful?

Last question: Is there anything else you want to add about your learning needs regarding eating disorders?
